# Characteristics of thermal images of the mammary gland and of performance in sows differing in health status and parity

**DOI:** 10.3389/fvets.2022.920302

**Published:** 2022-09-02

**Authors:** Stephan Rosengart, Bussarakam Chuppava, Lea-Sophie Trost, Hubert Henne, Jens Tetens, Imke Traulsen, Ansgar Deermann, Michael Wendt, Christian Visscher

**Affiliations:** ^1^Clinic for Swine and Small Ruminants, Forensic Medicine and Ambulatory Service, University of Veterinary Medicine Hannover, Foundation, Hanover, Germany; ^2^Institute for Animal Nutrition, University of Veterinary Medicine Hannover, Foundation, Hanover, Germany; ^3^Department of Animal Sciences, Livestock Systems, Georg-August-University Göttingen, Göttingen, Germany; ^4^BHZP GmbH, Dahlenburg-Ellringen, Germany; ^5^Department of Animal Sciences, University of Göttingen, Göttingen, Germany; ^6^EVH Select GmbH, Meppen, Germany

**Keywords:** infrared imaging, postpartum dysgalactia syndrome, precision farming, smart farming, healthcare, welfare monitoring, udder, early disease detection

## Abstract

Precision livestock farming can combine sensors and complex data to provide a simple score of meaningful productivity, pig welfare, and farm sustainability, which are the main drivers of modern pig production. Examples include using infrared thermography to monitor the temperature of sows to detect the early stages of the disease. To take account of these drivers, we assigned 697 hybrid (BHZP db. Viktoria) sows to four parity groups. In addition, by pooling clinical findings from every sow and their piglets, sows were classified into three groups for the annotation: healthy, clinically suspicious, and diseased. Besides, the udder was thermographed, and performance data were documented. Results showed that the piglets of diseased sows with eighth or higher parity had the lowest daily weight gain [healthy; 192 g ± 31.2, clinically suspicious; 191 g ± 31.3, diseased; 148 g ± 50.3 (*p* < 0.05)] and the highest number of stillborn piglets (healthy; 2.2 ± 2.39, clinically suspicious; 2.0 ± 1.62, diseased; 3.91 ± 4.93). Moreover, all diseased sows showed higher maximal skin temperatures by infrared thermography of the udder (*p* < 0.05). Thus, thermography coupled with Artificial Intelligence (AI) systems can help identify and orient the diagnosis of symptomatic animals to prompt adequate reaction at the earliest time.

## Introduction

Precision livestock farming (PLF) is key to optimizing farming processes (1, 2). PLF technologies are becoming increasingly important in modern pig production in terms of animal welfare and farm sustainability, with special regard to the survival rate and performance of healthy piglets after birth (1). Recent techniques based on thermal imaging allow estimating the body core temperature by measuring the surface temperature of different body parts without touching the animal (3–5). The temperature is determined indirectly *via* the radiation intensity (5). Previous studies reported using infrared thermography to assess the temperature rise for mastitis diagnosis in dogs, sheep, and cows (6–16) and to detect the disease in pig production (17, 18). In addition, infrared thermography was used in piglets. A correlation was found between the temperature of individual body regions (ear base and back) and rectal temperature (19), and between the temperature of individual body regions (eye, ear base, back, and anus) and the age and growth rate of the piglets (20). To reduce the mortality rate through illness or infection, early disease detection is an important monitor, especially in sows (18, 21). Monitoring of sows' health is the key to preventing and controlling diseases in sows, and it guarantees optimal rearing conditions for piglets (18).

Shortly after farrowing, sows can suffer from postpartum dysgalactia syndrome (PDS), which disturbs the sows' performance and impairs animal welfare (22, 23). The leading symptoms of PDS are a body temperature higher than 39.5°C and reduced feed intake (24). Furthermore, purulent vaginal discharge and inflammation with swelling and reddening of the mammary gland may occur (25). If the udder is painful, the sows rest on it more often without presenting it to the piglets (25–27). Therefore, piglets cannot access teats, and this will reduce their intake of colostrum and milk. Too little colostrum intake negatively impacts growth performance and reduces the survival chances of a suckling piglet until weaning (28). Moreover, milk intake and piglets' daily weight gain until weaning show a highly positive correlation (29). Many healthy piglets with a high daily weight gain can reasonably achieve satisfactory weight at weaning if suckling from sows with high colostrum and milk secretion. Through genetic selection and management improvement in recent years, this is more important than in the past because the number of live-born piglets per sow per year has increased (30, 31). In addition, the increase in litter variation due to large litters is an additional factor that minimizes survival rates of suckling piglets, especially of small suckling piglets (32) and especially in multiparous breed sows. High milk secretion can be expected in healthy sows reared under a favorable farming environment (management and feeding) for optimal expression of genetic potential for neuroendocrine support during gestation and lactation (23, 33–36). During the suckling period, daily weight gain of suckling piglets of about 200 g can then be expected (37). Moreover, when there is a lack of milk, the hungry piglets show restless behavior (33, 38), injuries to the carpal joints or face, and growth retardation (23). In addition, piglets born from PDS-affected sows are more prone to diarrhea, resulting in a higher mortality rate (23, 39). In order to keep sows healthy, it is important to identify and treat sows diagnosed with the disease as soon as possible (40).

As mentioned above, PDS is associated with an increase in core body temperature (35). Mastitis is generally understood to be inflammation of the parenchyma of the udder (26). Inflammation is characterized by redness, swelling, pain, heating, and loss of function (18, 41). In both cases, the increase in temperature is a very frequent symptom. Therefore, the results that using infrared thermography of the mammary gland, in general, can make a helpful contribution to finding diseased sows with poor milk production have been reported in numerous studies (18, 42, 43) because those animals have warmer temperatures in the thermal image of the mammary gland. Whether the age and parity of sows have an influence on the information contained in the thermal image of the mammary gland has not yet been researched. Furthermore, it is known that the age and parity of sows have an influence on the sows' performance (44–46). Therefore, we hypothesized that the temperature in the thermal image of the mammary gland and the information from the thermal images about performance and health status differ between parity groups.

Thus, the aim of this study was to examine whether there are certain parities or parity groups in which infrared thermography of the udder allows differentiation between PDS-affected sows and non-PDS-affected sows and between sows with high and low performance. In addition, whether there are parities or parity groups in which infrared thermography of the udder allows no differentiation regarding this.

## Materials and methods

### Animals and diets

Data collection and animal housing were carried out on a farm in Lower Saxony, Germany from August 2019 to November 2020 in accordance with German regulations, and the research protocol was approved by the Animal Welfare Officer of the University of Veterinary Medicine Hannover, Hanover, Germany (reference: TVO-2020-V-9). A total of 487 db, Viktoria hybrid sows (BHZP Landrace × BHZP Large White, Bundes Hybrid Zucht Programm (BHZP), Ellringen, Germany) with a parity ranging from 1 to 14 were used in this study. The sows were examined throughout up to three lactations, so a total of 697 sows were examined at birth.

The sows studied were kept in four identical farrowing units, each with 24 ProDromi farrowing pens (about 12 pens on each side). The four farrowing units were arranged behind each other. Sows were housed in farrowing crates. Antibiotic treatments were not administered to sows within at least seven days prior to farrowing. In compliance with the analysis, the nutritional content of the sows' diet is shown in Table 1. More detailed information has been previously described by Rosengart et al. (18).

**Table 1:** Nutrient contents in the gestation and lactation diet (pelleted complete feed) in accordance with the analysis (g/kg as fed).

**Item**	**Gestation diet**	**Lactation diet**
Dry matter	887 ± 7.5	889 ± 6.9
Crude protein	142 ± 3.4	163 ± 10.1
Crude fat	30 ± 1.5	36 ± 4.0
Crude fiber	72 ± 5.8	48 ± 3.7
Crude ash	53 ± 1.4	52 ± 3.0
Calcium	7.2 ± 1.0	8.1 ± 1.0
Phosphorus	4.8 ± 0.4	6 ± 0.2
Energy (MJ ME/kg)	11.7 ± 0.3	13 ± 0.3

The feed was designed in accordance with the recommendations of the Society for Nutritional Physiology (GfE) (47).

### Experimental procedure and sampling

Data acquisition from the sows and the piglets was performed as described by Rosengart et al. (18). Briefly, during the experimental period, a clinical examination and infrared thermography of the mammary gland were performed shortly after farrowing, ~14 days after farrowing and at weaning.

**Figure 1:**
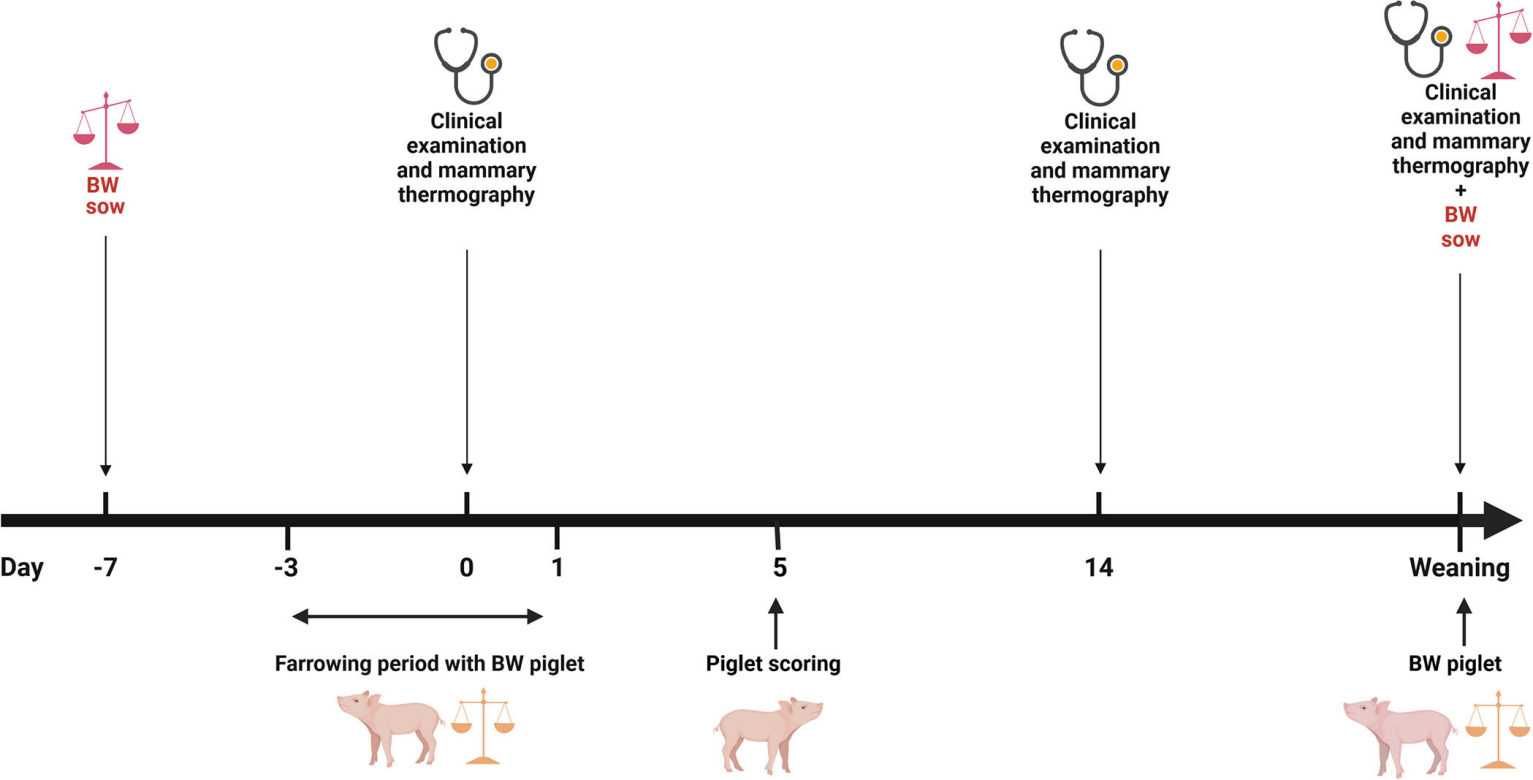
Overview of the data collection. BW, bodyweight; (Figure was created with BioRender.com). Adapted from Rosengart et al. (18).

A skin score of the carpal joints of the piglets took place about 5 days after birth (Figure 1). The modified scoring system from the Board of Trustees for Technology and Construction in Agriculture (Kuratorium für Technik und Bauwesen in der Landwirtschaft (KTBL)) (48) was used. Briefly, a score of 0 meant no bloody or encrusted injuries on the carpal joints with a diameter of 0.5 cm or more; a score of 1 meant >50% of the litter with bloody or encrusted injuries on the carpal joints with a diameter of 0.5 cm or more; and score 2 meant more than 50% of the litter with bloody or encrusted injuries on the carpal joints with a diameter of 0.5 cm or more (18).

### Readings

#### Sows' and piglets' performance

The piglets were individually marked and weighed within the first 24 h of farrowing. In addition, the piglets were weighed at weaning. Thus, the daily weight gain of each piglet could be determined during this period. Usually, cross-fostering took place after the first 24 h of farrowing. Furthermore, all sows were weighed twice. The first weighing took place when sows were moved to the farrowing pen and the second weighing occurred when they were removed from the farrowing pen (Figure 1). Sows were moved to the farrowing pen on approximately day 110 of gestation. Weighing was performed with a pair of low floor scales (Meier-Brakenberg GmbH & Co. KG, Extertal, Germany). The scales were designed for a weight range from 0 to 2000 kg and weighed in increments of 1 kg. Moreover, all collected data for the sow management software db. planer (Version 1906, BHZP GmbH, Dahlenburg-Ellringen, Germany) were available, i.e., sows' parity, a total of born piglets (TBP), number of stillborn piglets (NS), number of piglets born alive (NBA), number of piglets that had died before weaning (PWM), and number of weaned piglets (NWP) for each sow for that litter.

#### Thermal image capture and analysis and diagnosis of PDS

The ambient temperature in the farrowing unit was read and recorded as displayed by the barn equipment before entering and after leaving a farrowing unit. The temperature measurement of the barn equipment was verified by non-stop temperature measurement with TGP-4500 Tinytag Plus 2 temperature loggers. Humidity was measured by Tinytag Plus 2 temperature loggers.

Every mammary complex was inspected individually shortly after birth (0, 1, or 2 days afterwards), ~14 days after birth, and at weaning. Inspection and palpation of the mammary gland characterized the clinical examination. The scoring system from previous studies (18, 42) was used for this. In this way, the degree of formation, the degree of redness, the consistency, whether there were nodes in the parenchyma, and whether mammary complexes were painful were described. Score 0 meant that everything was physiological, score 2 meant the greatest deviation from normal, and score 1 was in the middle of both. Clinical findings were recorded. Moreover, sows with no food consumption, purulent discharge, or that were reluctant to stand up were recorded, too.

**Figure 2:**
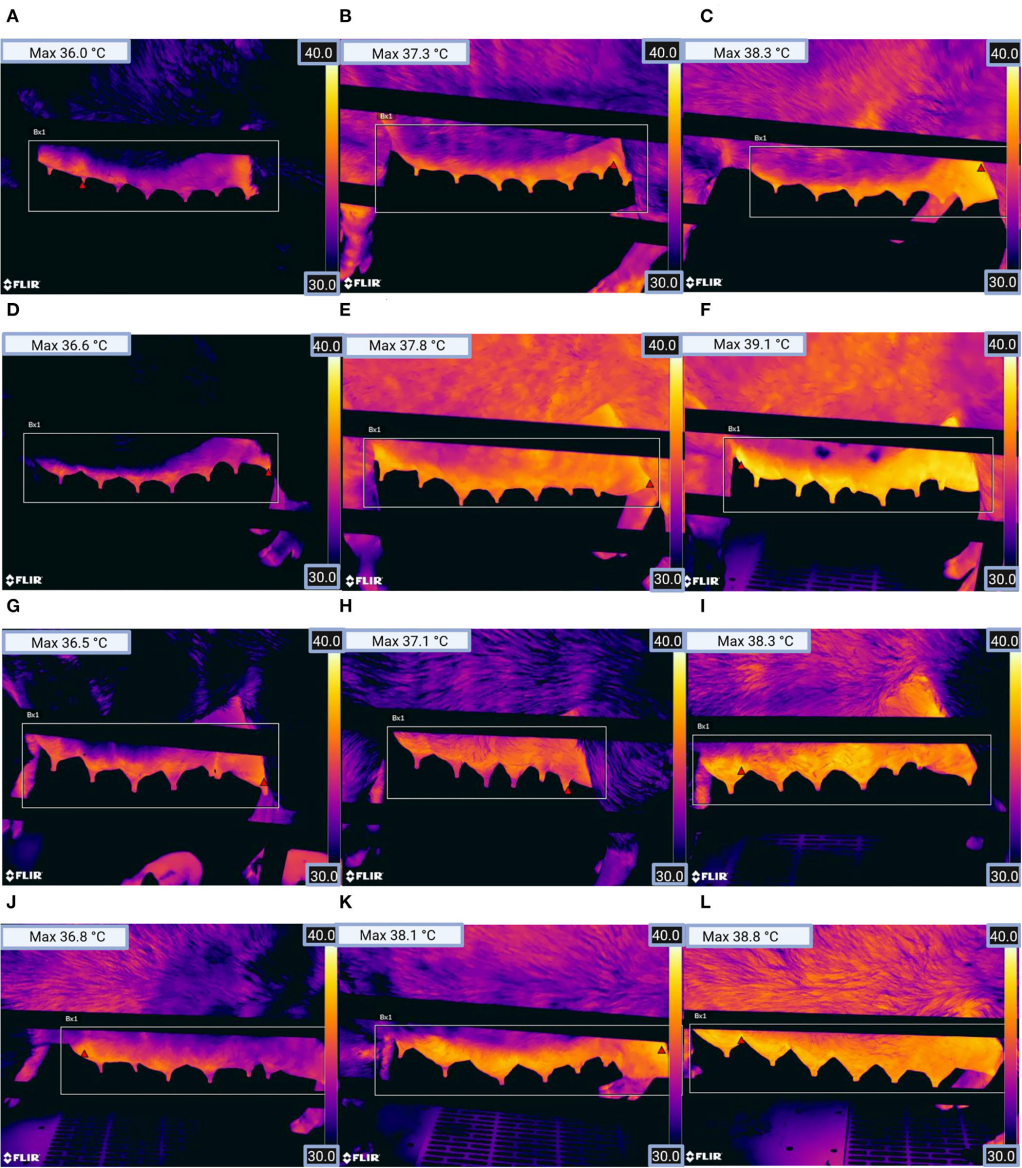
Thermographic images from the left mammary gland of sows for **(A)** parity group 1 (healthy), **(B)** parity group 1 (clinically suspicious), **(C)** parity group 1 (diseased), **(D)** parity group 2 (healthy), **(E)** parity group 2 (clinically suspicious), **(F)** parity group 2 (diseased), **(G)** parity group 3 (healthy), **(H)** parity group 3 (clinically suspicious), **(I)** parity group 3 (diseased), **(J)** parity group 4 (healthy), **(K)** parity group 4 (clinically suspicious), and **(L)** parity group 4 (diseased). The red triangle within the rectangle in the image shows the location of the pixel with the highest temperature (see Max temperature top left in the picture). (Figure was created with BioRender.com).

Following the clinical examination of the sows, a picture with infrared thermography was taken of the sows' mammary gland [on both sides of the mammary gland, Figure 2 (FLIR T540, FLIR Systems, Inc., Wilsonville, or, the USA)]. Infrared images of the sows, with a temperature scale, were taken in a standing position. In accordance with previous studies (42, 49), emissivity was set at 0.96. The spacing between the sow and the camera was about one meter and was measured by the camera before each image was captured. While capturing an image, an angle of <60° to the udder was avoided (42). The farrowing pens were ProDromi farrowing pens, so the heating plates of the piglet nests were located in front of the sows' head and surrounded by plastic. That is why stray radiation from that heating was no problem. Lastly, with a Veterinary Thermometer VT 1831 (Microlife AG, Widnau, Switzerland), the rectal temperature was measured and recorded.

The evaluation of the thermal images was performed with the FLIR Tools program (FLIR Systems, Inc.). Using this program, the highest skin temperature of each thermal image could be recorded. This way was gone following a previous study (43) that achieved excellent results with this analysis method. Subsequently, the mean value of the highest skin temperature on the right and left sides of the udder was calculated.

**Figure 3:**
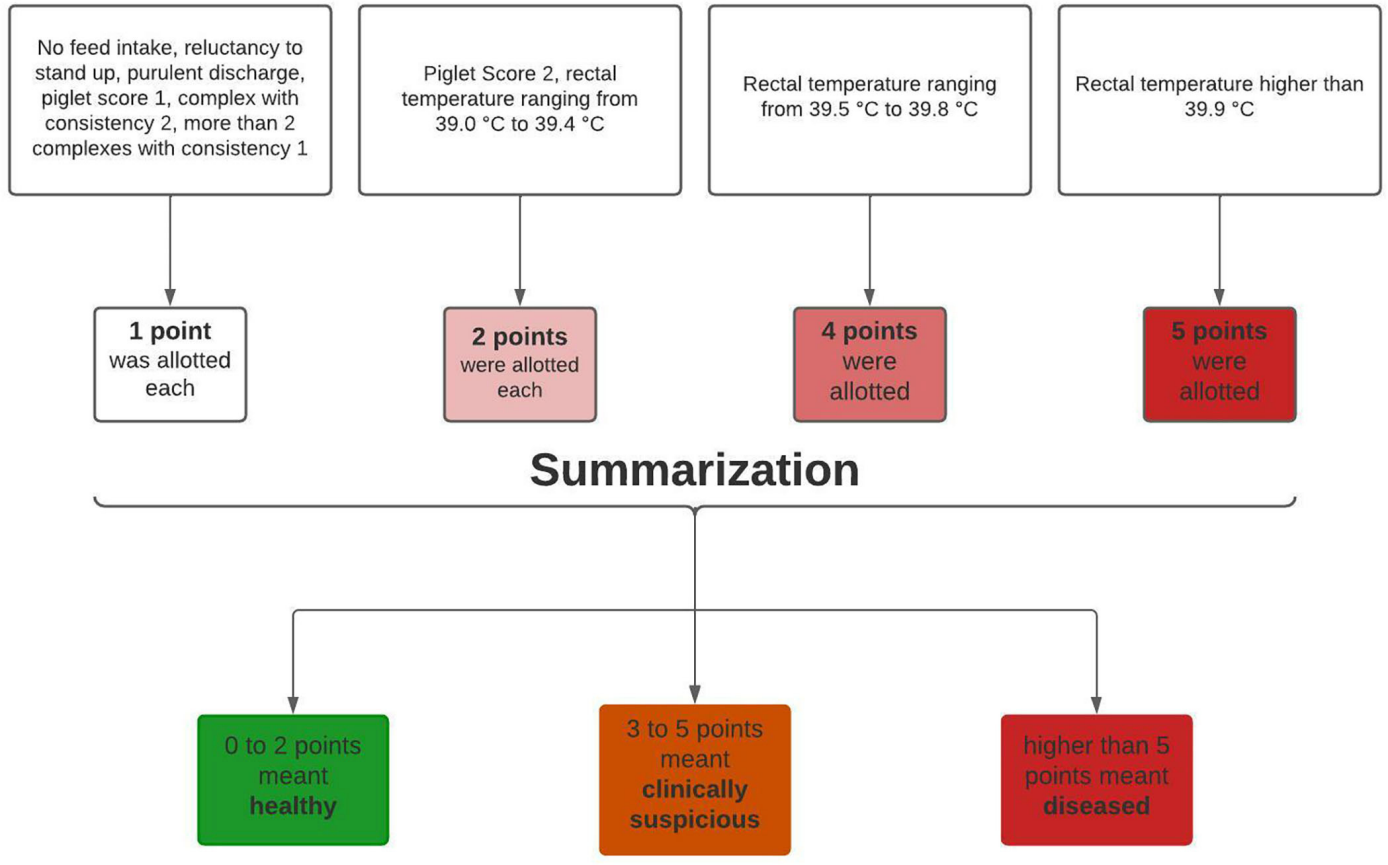
Schematic diagram about aggregation of clinical data. Adapted from Rosengart et al. (18).

To detect PDS-affected sows, the clinical data were afterwards aggregated into a health score (18) depending on the extent of clinical findings, where the number of score points reflected a weighted number of clinical findings. No feed intake, reluctance to stand up, purulent discharge, piglet score 1, mammary complex with consistency two or more than two mammary complexes with consistency 1 meant one point was allotted. A piglet score of 2 or a rectal temperature ranging from 39 to 39.4°C meant two points were allotted. A rectal temperature ranging from 39.5 to 39.8°C meant a score of four points, and a rectal temperature higher than 39.8°C meant a score of five points (Figure 3).

**Figure 4:**
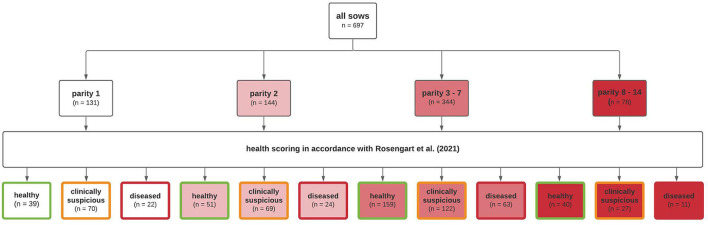
Schematic diagram showing group categorization of investigated sows.

A healthy group scored a total of zero to two points, a clinically suspicious group a total of three to five points, and a total of more than five given points meant classification into the diseased group. In addition, the sows were categorized according to parity. By categorizing according to parity and health status, all sows were divided into 12 groups (Figure 4). The first group categorization was made at parity. First parity sows were allocated to parity group 1 (*n* = 131), second parity sows to parity group 2 (*n* = 144), third to seventh parity sows to parity group 3 (*n* = 344), and sows with eighth or higher parity to parity group 4 (*n* = 78). Moreover, every parity group was divided into three health classes (healthy, clinically suspicious, and diseased) (Figures 3, 4).

### Statistical methods

Data were statistically analyzed using the SAS Enterprise Guide (version 7.1, Fa. SAS Institute Inc., Cary, NC, USA). Mean values and the standard deviation were calculated for all parameters shown in Tables 1, 2, **7**. The Kolmogorov-Smirnov test was used to test for normal data distribution. Interactions between health categories and parity groups were tested with a two-way analysis of variance (ANOVA). Differences between health categories and parity groups were examined using a one-way analysis of variance (ANOVA). For multiple pairwise means comparison between the three groups, the Ryan-Einot-Gabriel-Welsch multiple-range test (REGWQ) was used. The data for which the correlations are given in Tables 3–**6** were all normally distributed. Therefore, the correlation coefficient was determined in accordance with Pearson. In accordance with Akoglu (50), 0.00–0.29 meant poor correlation, 0.3–0.59 fair correlation, 0.6–0.79 moderate correlation, 0.8–0.99 very strong correlation, and one perfect correlation.

**Table 2:** Thermographic skin temperature and rectal temperature (°C) compared between healthy, clinically suspicious and diseased sows of different parities.

**Item**	**Parity**	***N***	**Healthy**	***N***	**Clinically suspicious**	***N***	**Diseased**
TH0	1	39	37.0^Aa^ ± 0.63	70	37.5^Ab^ ± 0.58	22	38.1^c^ ± 0.52
TH0	2	51	37.3^Ba^ ± 0.49	69	37.6^ABb^ ± 0.48	24	38.5^c^ ± 0.59
TH0	3-7	159	37.2^ABa^ ± 0.53	122	37.7^Bb^ ± 0.54	63	38.4^c^ ± 0.57
TH0	8+	40	37.1^ABa^ ± 0.52	27	37.5^ABb^ ± 0.53	11	38.3^c^ ± 0.39
RT0	1	39	38.9^Aa^ ± 0.24	70	39.2^b^ ± 0.22	22	39.7^Ac^ ± 0.36
RT0	2	51	38.9^Aa^ ± 0.29	69	39.3^b^ ± 0.23	24	40.2^Bc^ ± 0.38
RT0	3-7	159	38.7^Ba^ ± 0.28	122	39.2^b^ ± 0.22	63	39.9^ABc^ ± 0.45
RT0	8+	40	38.7^Ba^ ± 0.31	27	39.2^b^ ± 0.27	11	39.8^Ac^ ± 0.26
TH14	1	37	37.8^A^ ± 0.60	68	38.0^A^ ± 0.50	21	38.0^A^ ± 0.50
TH14	2	48	37.6^ABa^ ± 0.54	63	37.8^ABa^ ± 0.47	23	38.0^Ab^ ± 0.58
TH14	3-7	149	37.6^ABa^ ± 0.62	115	37.7^BCab^ ± 0.64	62	37.8^Ab^ ± 0.66
TH14	8+	36	37.4^B^ ± 0.61	26	37.5^C^ ± 0.66	8	37.0^B^ ± 0.83
RT14	1	38	39.3^A^ ± 0.33	68	39.3^A^ ± 0.38	21	39.4^A^ ± 0.22
RT14	2	48	39.0^B^ ± 0.35	63	39.1^B^ ± 0.37	23	39.1^B^ ± 0.35
RT14	3-7	150	38.9^BC^ ± 0.32	115	38.9^C^ ± 0.31	62	39.0^B^ ± 0.3
RT14	8+	36	38.8C^a^ ± 0.29	26	38.9^Ca^ ± 0.44	9	38.5^Cb^ ± 0.22
TH21	1	36	37.5^Aa^ ± 0.61	66	37.8^Aab^ ± 0.52	21	37.9^Ab^ ± 0.62
TH21	2	48	37.3^ABa^ ± 0.60	65	37.4^Ba^ ± 0.46	23	37.7^ABb^ ± 0.51
TH21	3-7	144	37.2^Ba^ ± 0.62	113	37.3^BCab^ ± 0.62	58	37.4^BCb^ ± 0.63
TH21	8+	36	36.8^C^ ± 0.59	24	37.0^C^ ± 0.83	9	37.1^C^ ± 0.71
RT21	1	36	38.9^Aa^ ± 0.38	66	39.0^Aab^ ± 0.41	21	39.2^Ab^ ± 0.63
RT21	2	48	38.6^B^ ± 0.31	65	38.7^B^ ± 0.33	23	38.8^B^ ± 0.25
RT21	3-7	144	38.4^Ca^ ± 0.35	113	38.5^Cb^ ± 0.36	58	38.6^Bb^ ± 0.35
RT21	8+	36	38.2^D^ ± 0.38	24	38.5^C^ ± 0.52	9	38.5^B^ ± 0.28

Mean value of the highest skin temperature of the udder (infrared thermography of the right and left side) shortly after farrowing (TH0), around 14 days after farrowing (TH14) and at weaning (TH21). Rectal temperature shortly after farrowing (RT0), around 14 days after farrowing (RT14) and at weaning (RT21).

^*a, b, c*^Values within a row with different superscripts differ significantly (*p* < 0.05).

^*A, B, C, D*^Values within a column with different superscripts differ significantly (*p* < 0.05).

**Table 3:** Pearson correlation coefficient between thermographic skin temperature, rectal temperature, stillborn piglets, number of weaned piglets and daily weight gain of suckling piglets of first parity sows.

***p*-value (*N*)**	**Pearson correlation coefficient**								
	**TH0.1**	**RT0.1**	**TH14.1**	**RT14.1**	**TH21.1**	**RT21.1**	**NS1**	**NWP1**	**DWG1**
TH0.1		**0.64**	**0.42**	0.15	**0.39**	**0.30**	−0.003	0.14	**−0.25**
RT0.1	**<0.0001**, ***n*** **=** **131**		0.21	0.13	**0.24**	0.15	−0.05	0.02	−0.13
TH14.1	**<0.0001**, ***n*** **=** **126**	<0.05, *n* = 126		**0.61**	**0.64**	**0.41**	−0.06	0.13	−0.18
RT14.1	0.09, *n* = 127	0.16, *n* = 127	**<0.0001**, ***n*** **=** **126**		**0.39**	**0.43**	−0.13	0.09	−0.14
TH21.1	**<0.0001**, ***n*** **=** **123**	**<0.01**, ***n*** **=** **123**	**<0.0001**, ***n*** **=** **122**	**<0.0001**, ***n*** **=** **123**		**0.71**	−0.06	0.06	**−0.19**
RT21.1	**<0.01**, ***n*** **=** **123**	0.09, *n* = 123	**<0.0001**, ***n*** **=** **122**	**<0.0001**, ***n*** **=** **123**	**<0.0001**, ***n*** **=** **123**		−0.01	0.12	**−0.28**
NS1	0.98, *n* = 131	0.54, *n* = 131	0.49, *n* = 126	0.13, *n* = 127	0.53, *n* = 123	0.90, *n* = 123		0.09	0.02
NWP1	0.12, *n* = 131	0.80, *n* = 131	0.15, *n* = 126	0.33, *n* = 127	0.51, *n* = 123	0.17, *n* = 123	0.32, *n* = 131		**−0.32**
DWG1	**<0.01**, ***n*** **=** **121**	0.14, *n* = 121	0.05, *n* = 116	0.12, *n* = 117	**<0.05**, ***n*** **=** **113**	**<0.01**, ***n*** **=** **113**	0.79, *n* = 121	**<0.01**, ***n*** **=** **121**	

Mean value of the highest skin temperature of the udder (infrared thermography of the right and the left side) of first parity sows shortly after farrowing (TH0.1), around 14 days after farrowing (TH14.1) and at weaning (TH21.1); rectal temperature of first parity sows shortly after farrowing (RT0.1), around 14 days after farrowing (RT14.1) and at weaning (RT21.1); NS1 (n); stillborn piglets of first parity sows, NWP1 (n); weaned piglets of first parity sows, DWG1 in g; daily weight gain of the piglets from farrowing to the 2nd weighing suckled by first parity sows. Values in bold indicate significant values with p < 0.05.

## Results

In general, there were no interactions between health categories and parity groups, except for the daily weight gain of the piglets (DWG), the rectal temperature shortly after birth (RT0), and the rectal temperature 14 days after birth (RT14).

### Infrared thermography and rectal temperature

Moreover, at three different time points (shortly after farrowing, 14-d postpartum, and at weaning), rectal temperature (RT) and the highest skin temperature of the sow's mammary gland were measured using thermography (TH) from the right and left sides of the udder. The mean value of the two measured values was calculated. Results with regard to parity and the three health condition groups of sows are shown in Table 2.

#### Readings shortly after farrowing

Shortly after farrowing, the diseased animals always showed the highest values in infrared thermography (TH0) and rectal temperature (RT0). Differences between health categories (healthy, clinically suspicious, and diseased) were statistically significant in every parity group (Table 2, *p* < 0.05). Age-dependent differences were marginal at this particular time (Table 2). The highest values were found in the diseased group of second parity sows (RT0.2, Table 2).

#### Readings 14 days postpartum and at weaning

On day 14, postpartum, diseased sows showed no differences or slightly elevated temperatures (parity 2 and 3–7; *p* < 0.05) in infrared thermography (TH14) in comparison to suspicious or healthy sows. With regard to rectal temperature (RT14), diseased sows showed no differences or lower values (parity 8+; *p* < 0.05) in rectal temperatures in comparison to suspicious or healthy sows. At weaning, the same tendency was seen in diseased sows, these animals showing no differences or slightly elevated TH21 (parity 1; 2; 3–7; *p* < 0.05) and RT21 values (parity 1; 3–7; *p* < 0.05) mainly in comparison to the healthy group (Table 2).

On day 14 after farrowing and weaning, sows showed an age-dependent regular decrease in TH and RT from first parity sows to the oldest ones (*p* < 0.05). This proved the case for all three different health status groups.

### Correlations

Table 3 shows the Pearson correlation coefficient between two variables from nine investigated parameters of first parity sows. Moderate correlations occurred between the TH21.1 and the RT21.1, the TH14.1 and the TH21.1, the TH0.1 and the RT0.1 and the TH14.1 and the RT14.1 (*p* < 0.0001).

Table 4 shows the Pearson correlation coefficient between two variables from nine investigated parameters of second parity sows. A moderate correlation occurred between the TH14.2 and the TH21.2 and the TH0.2 and the RT0.2 *p* < 0.0001).

**Table 4:** Pearson correlation coefficient between thermographic skin temperature, rectal temperature, stillborn piglets, number of weaned piglets and daily weight gain of suckling piglets of second parity sows.

***p*-value (*N*)**	**Pearson correlation coefficient**								
	**TH0.2**	**RT0.2**	**TH14.2**	**RT14.2**	**TH21.2**	**RT21.2**	**NS2**	**NWP2**	**DWG2**
TH0.2		**0.67**	**0.47**	**0.23**	**0.47**	**0.31**	−0.01	0.11	−0.16
RT0.2	**<0.0001**, ***n*** **=** **144**		0.20	0.17	0.21	0.21	0	0.06	0
TH14.2	**<0.0001**, ***n*** **=** **134**	<0.05, *n* = 134		**0.53**	**0.77**	**0.43**	−0.16	**0.20**	−0.17
RT14.2	**<0.01**, ***n*** **=** **134**	<0.05, *n* = 134	**<0.0001**, ***n*** **=** **134**		**0.40**	**0.42**	0.02	**0.25**	−0.16
TH21.2	**<0.0001**, ***n*** **=** **136**	<0.05, *n* = 136	**<0.0001**, ***n*** **=** **133**	**<0.0001**, ***n*** **=** **133**		**0.59**	**−0.18**	**0.22**	−0.05
RT21.2	**<0.01**, ***n*** **=** **136**	<0.05, *n* = 136	**<0.0001**, ***n*** **=** **133**	**<0.0001**, ***n*** **=** **133**	**<0.0001**, ***n*** **=** **136**		**−0.21**	0.17	−0.15
NS2	0.90, *n* = 144	1, *n* = 144	0.07, *n* = 134	0.78, *n* = 134	**<0.05**, ***n*** **=** **136**	**<0.05**, ***n*** **=** **136**		−0.15	0.18
NWP2	0.21, *n* = 144	0.45, *n* = 144	**<0.05**, ***n*** **=** **134**	**<0.01**, ***n*** **=** **134**	**<0.05**, ***n*** **=** **136**	0.05, *n* = 136	0.08, *n* = 144		**−0.30**
DWG2	0.10, *n* = 100	0.97, *n* = 100	0.10, *n* = 94	0.13, *n* = 94	0.63, *n* = 95	0.14, *n* = 95	0.07, *n* = 100	**<0.01**, ***n*** **=** **100**	

Mean value of the highest skin temperature of the udder (infrared thermography of the right and left side) of second parity sows shortly after farrowing (TH0.2), around 14 days after farrowing (TH14.2) and at weaning (TH21.2); rectal temperature of second parity sows shortly after farrowing (RT0.2), around 14 days after farrowing (RT14.2) and at weaning (RT21.2); NS2 (n); stillborn piglets of second parity sows, NWP2 (n); weaned piglets of second parity sows, DWG2 in g; daily weight gain of the piglets from farrowing to the 2nd weighing suckled by second parity sows. Values in bold indicate significant values with p <0.05.

Table 5 shows the Pearson correlation coefficient between two variables from nine investigated parameters of third to seventh parity sows. A moderate correlation occurred between the TH14.3-7 and the TH21.3-7, the TH0.3-7 and the RT0.3-7, and the TH21.3-7 and the RT21.3-7 (*p* < 0.0001).

**Table 5:** Pearson correlation coefficient between thermographic skin temperature, rectal temperature, stillborn piglets, number of weaned piglets and daily weight gain of suckling piglets of third to seventh parity sows.

***p*-value (*N*)**	**Pearson correlation coefficient**								
	**TH0.3**	**RT0.3**	**TH14.3**	**RT14.3**	**TH21.3**	**RT21.3**	**NS3**	**NWP3**	**DWG3**
TH0.3-7		**0.73**	**0.46**	**0.22**	**0.42**	**0.30**	−0.03	0	−0.09
RT0.3-7	**<0.0001**, ***n*** **=** **344**		**0.17**	0.18	0.14	**0.19**	−0.06	−0.03	−0.04
TH14.3-7	**<0.0001**, ***n*** **=** **326**	**<0.01**, ***n*** **=** **325**		**0.49**	**0.75**	**0.40**	−0.03	0.05	0.02
RT14.3-7	**<0.0001**, ***n*** **=** **327**	<0.05, *n* = 326	<0.0001, *n* = 326		**0.33**	**0.44**	**−0.15**	0.02	−0.07
TH21.3-7	**<0.0001**, ***n*** **=** **315**	<0.05, *n* = 314	**<0.0001**, ***n*** **=** **310**	**<0.0001**, ***n*** **=** **311**		**0.62**	−0.03	0.08	0
RT21.3-7	**<0.0001**, ***n*** **=** **315**	**<0.01**, ***n*** **=** **314**	**<0.0001**, ***n*** **=** **310**	**<0.0001**, ***n*** **=** **311**	**<0.0001**, ***n*** **=** **315**		−0.03	0.07	**−0.13**
NS3-7	0.52, *n* = 345	0.27, *n* = 344	0.58, *n* = 326	**<0.01**, ***n*** **=** **327**	0.65, *n* = 315	0.57, *n* = 315		0.01	0.03
NWP3-7	0.96, *n* = 345	0.61, *n* = 344	0.40, *n* = 326	0.73, *n* = 327	0.17, *n* = 315	0.25, *n* = 315	0.86, *n* = 345		−0.06
DWG3-7	0.16, *n* = 265	0.57, *n* = 264	0.71, *n* = 252	0.25, *n* = 253	0.91, *n* = 244	**<0.05**, ***n*** **=** **244**	0.68, *n* = 265	0.29, *n* = 265	

Mean value of the highest skin temperature of the udder (infrared thermography of the right and left side) of third to seventh parity sows shortly after farrowing (TH0.3-7), around 14 days after farrowing (TH14.3-7) and at weaning (TH21.3-7); rectal temperature of third to seventh parity sows shortly after farrowing (RT0.3-7), around 14 days after farrowing (RT14.3-7) and at weaning (RT21.3-7); NS3-7 (n); stillborn piglets of third to seventh parity sows, NWP3-7 (n); weaned piglets of third to seventh parity sows, DWG3-7 in g; daily weight gain of the piglets from farrowing to the 2nd weighing suckled by third to seventh parity sows. Values in bold indicate significant values with p < 0.05.

Table 6 shows the Pearson correlation coefficient between two variables from nine investigated parameters, from eighth and higher parity sows. Moderate correlations occurred between the TH21.8 + and the RT21.8 +, the TH0.8 + and the RT0.8 +, the TH14.8 + and the TH21.8 + and the TH14.8 + and the RT14.8 + (*p* < 0.0001).

**Table 6:** Pearson correlation coefficient between thermographic skin temperature, rectal temperature, stillborn piglets, number of weaned piglets and daily weight gain of suckling piglets of eighth and higher parity sows.

***p*-value (*N*)**	**Pearson correlation coefficient**								
	**TH0.8+**	**RT0.8+**	**TH14.8+**	**RT14.8+**	**TH21.8+**	**RT21.8+**	**NS8+**	**NWP8+**	**DWG8+**
TH0.8+		**0.71**	0.17	0	**0.38**	**0.33**	0.03	−0.11	**−0.32**
RT0.8+	**<0.0001**, ***n*** **=** **78**		−0.04	−0.06	0.16	0.25	0.01	−0.08	**−0.30**
TH14.8+	0.16, *n* = 70	0.72, *n* = 70		**0.64**	**0.71**	**0.39**	**−0.26**	0.08	0.18
RT14.8+	1.00, *n* = 71	0.62, *n* = 71	**<0.0001**, ***n*** **=** **70**		**0.50**	**0.45**	−0.21	0.23	0.03
TH21.8+	**<0.01**, ***n*** **=** **69**	0.18, *n* = 69	**<0.0001**, ***n*** **=** **68**	**<0.0001**, ***n*** **=** **68**		**0.75**	−0.07	−0.03	0.08
RT21.8+	**<0.01**, ***n*** **=** **69**	<0.05, *n* = 69	**<0.01**, ***n*** **=** **68**	**0.0001**, ***n*** **=** **68**	**<0.0001**, ***n*** **=** **69**		0.04	0.11	−0.15
NS8+	0.81, *n* = 78	0.90, *n* = 65	**<0.05**, ***n*** **=** **70**	0.08, *n* = 71	0.59, *n* = 69	0.73, *n* = 69		−0.12	**−0.28**
NWP8+	0.35, *n* = 78	0.50, *n* = 78	0.53, *n* = 70	0.05, *n* = 71	0.79, *n* = 69	0.37, *n* = 69	0.29, *n* = 78		−0.15
DWG8+	**<0.01**, ***n*** **=** **72**	**<0.05**, ***n*** **=** **72**	0.15, *n* = 65	0.83, *n* = 65	0.54, *n* = 64	0.22, *n* = 64	**<0.05**, ***n*** **=** **72**	0.21, *n* = 72	

Mean value of the highest skin temperature of the udder (infrared thermography of the right and left side) of eighth and higher parity sows shortly after farrowing (TH0.8+), around 14 days after farrowing (TH14.8+) and at weaning (TH21.8+); rectal temperature of eighth and higher parity sows shortly after farrowing (RT0.8+), around 14 days after farrowing (RT14.8+) and at weaning (RT21.8+); NS8+ (n); stillborn piglets of eighth and higher parity sows, NWP8+ (n); weaned piglets of eighth and higher parity sows, DWG8+ in g; daily weight gain of the piglets from farrowing to the 2nd weighing suckled by eighth and higher parity sows. Values in bold indicate significant values with p < 0.05.

### Sows' and piglets' performance

#### Differences between the parity groups

##### Healthy sows

The results of the evaluations of the performance parameters of sows in relation to health and parity and their piglets are presented in Table 7. The parity group with sows from third to seventh parity in the diseased group showed a significantly lower number of total born piglets (TBP3-7, *p* < 0.05) as well as a significantly lower number of piglets born alive (NBA3-7, *p* < 0.05). The parity group with sows equaling or more than eight parities (≥8) was most often characterized by significant differences. This group showed significant differences between the three health categories in the number of piglets born alive (NBA8+, *p* < 0.05). In addition, the same parity group showed a significantly higher weaning weight of the sows (SW2.8+, *p* < 0.05) in the diseased group compared with the healthy group. However, weight loss during lactation did not differ. Piglets suckled by old sows in the diseased group had a significantly lower daily weight gain (DWG8+, *p* < 0.05). Additionally, piglets suckled by diseased first parity sows also showed a significantly lower daily weight gain (DWG1, *p* < 0.05).

**Table 7:** Comparison of performance parameters between healthy, clinically suspicious and diseased sows of different parities.

**Item**	**Parity**	***N***	**Healthy**	***N***	**Clinically suspicious**	***N***	**Diseased**
TBP	1	39	17.6^A^ ± 2.8	70	17.2 ± 3.54	22	16.4 ± 3.79
TBP	2	51	17.4^A^ ± 2.88	69	17.3 ± 3.20	24	16.6 ± 3.34
TBP	3–7	159	17.8^Aa^ ± 3.36	122	18.0^a^ ± 3.25	64	16.6^b^ ± 3.73
TBP	8+	40	14.9^B^ ± 3.16	27	16.8 ± 2.78	11	14.5 ± 4.23
NBA	1	39	17.3^A^ ± 2.93	70	16.9^A^ ± 3.36	22	16.1^A^ ± 3.7
NBA	2	51	16.9^A^ ± 2.92	69	16.9^A^ ± 3.08	24	16.2^A^ ± 3.47
NBA	3–7	159	16.7^Aa^ ± 3.3	122	16.9^Aa^ ± 3.4	64	15.4^Ab^ ± 3.89
NBA	8+	40	12.7^Ba^ ± 2.93	27	14.8^Bb^ ± 2.73	11	10.6^Bc^ ± 3.17
NS	1	39	0.33^A^ ± 0.74	70	0.3^A^ ± 0.67	22	0.23^A^ ± 0.69
NS	2	51	0.51^AB^ ± 0.92	69	0.33^A^ ± 0.72	24	0.42^A^ ± 0.97
NS	3–7	159	1.12^B^ ± 1.49	122	1.09^B^ ± 2.15	64	1.2^A^ ± 2.27
NS	8+	40	2.2^C^ ± 2.39	27	2.0^C^ ± 1.62	11	3.91^B^ ± 4.93
PWM	1	37	3.28^A^ ± 2.73	70	2.99 ± 2.16	22	3.23^A^ ± 2.31
PWM	2	51	2.59^AB^ ± 2.69	69	2.62 ± 2.18	24	2.04^AB^ ± 1.94
PWM	3–7	159	1.84^BC^ ± 2.02	122	2.05 ± 1.97	64	1.72^AB^ ± 2.11
PWM	8+	40	1.33^C^ ± 1.21	27	1.96 ± 2.89	11	0.82^B^ ± 1.08
NWP	1	39	12.4^A^ ± 1.51	70	11.9 ± 2.84	22	13.0^A^ ± 2.36
NWP	2	51	12.6^A^ ± 3.4	69	12.6 ± 1.89	24	13.1^A^ ± 2.85
NWP	3–7	159	12.1^AB^ ± 1.33	122	12 ± 1.75	64	12.1^A^ ± 1.73
NWP	8+	40	11.4^B^ ± 1.42	27	11.6 ± 1.78	11	10.5^B^ ± 2.66
SW1	1	38	236^A^ ± 19.3	70	233^A^ ± 17.5	22	237^A^ ± 16.3
SW1	2	50	241^A^ ± 15.5	66	244^B^ ± 21.8	24	242^A^ ± 13.7
SW1	3–7	152	274^B^ ± 26.1	121	276^C^ ± 26.5	63	275^B^ ± 19.5
SW1	8+	40	304^C^ ± 21.7	27	320^D^ ± 28.9	10	320^C^ ± 24
SW2	1	37	181^A^ ± 18.1	70	181^A^ ± 14.2	22	185^A^ ± 15.8
SW2	2	50	199^B^ ± 20.3	66	202^B^ ± 18.8	24	201^B^ ± 15.6
SW2	3–7	152	229^C^ ± 24.3	120	229^C^ ± 26.8	63	230^C^ ± 19.7
SW2	8+	39	265^Da^ ± 16.9	27	277^Dab^ ± 22.2	10	284^Db^ ± 23.4
SWD	1	37	55.7^A^ ± 13.4	70	51.9^A^ ± 13.2	22	52.0^A^ ± 17.0
SWD	2	50	42.5^B^ ± 16.6	66	42.4^B^ ± 14.4	24	40.6^AB^ ± 13.1
SWD	3–7	152	45.0^B^ ± 17.5	120	46.7^AB^ ± 18.3	63	45.0^AB^ ± 16.2
SWD	8+	39	38.4^B^ ± 13.6	27	43.1^B^ ± 17.8	10	36.9^B^ ± 12.0
DWG	1	35	197^a^ ± 22.9	64	187^a^ ± 29.3	22	171^ABb^ ± 33.8
DWG	2	38	185 ± 35.3	46	187 ± 33.7	16	191^A^ ± 27.2
DWG	3–7	116	197 ± 33.4	100	194 ± 35.6	49	193^A^ ± 35.5
DWG	8+	37	192^a^ ± 31.2	25	191^a^ ± 31.3	10	148^Bb^ ± 50.3

TBP (n); total number of born piglets, NBA (n); piglets born alive, NS (n); stillborn piglets, PWM (n); pre-weaning mortality, NWP (n); weaned piglets, SW1 (kg); sow weight when entering the farrowing house, SW2 (kg); sow weight at weaning, SWD (kg); weight loss during lactation (SW1-SW2), DWG (g); daily weight gain of the piglets from farrowing to the 2nd weighing.

^*a, b, c*^Values within a row with different superscripts differ significantly (p < 0.05).

^*A, B, C, D*^Values within a column with different superscripts differ significantly (p < 0.05).

##### Clinically suspicious sows

Clinically suspicious sows in the parity group with sows equaling or more than eight parities (≥8) stood out again with significant differences. Likewise, the clinically suspicious sows showed a significantly lower number of piglets born alive in the group of highest parity sows (NBA8+, Table 7, *p* < 0.05). Moreover, the same sows had significantly more stillborn piglets (NS8+, *p* < 0.05) compared to first parity sows (NS1, Table 7). Again, the weight of sows entering the farrowing room (SW1) and also the weaning weight of sows (SW2) were lower for the first parity and the highest for sows in the eighth or higher parity (Table 7, *p* < 0.05). However, first parity sows again had the greatest weight loss during lactation (SWD1, Table 7).

##### Diseased sows

Diseased sows in the parity group with sows equaling or more than eight parities (8+) stood out with significant differences. Likewise, the diseased sows showed a significantly lower number of piglets born alive in the group with the highest parity sows (NBA8 +, Table 7, *p* < 0.05)). In addition, the same sows weaned significantly fewer piglets (NWP) than the other sows (Table 7, *p* < 0.05). Moreover, the highest parity group sows had a significantly higher number of stillborn piglets (NS8 +, Table 7, *p* < 0.05). Pre-weaning mortality (PWM) was highest in litters of sows in first parity and the lowest number of piglets that died was documented in the litters of eighth or higher parity sows (Table 7, *p* < 0.05). Again, the weight of sows entering the farrowing room (SW1) and the weight at the weaning of sows (SW2) were lowest for first parity sows and highest for eighth and higher parity sows (Table 7, *p* < 0.05). However, first parity sows again had the greatest weight loss during lactation, while eight and higher parity sows had the lowest (SWD1, Table 7, *p* < 0.05). Moreover, piglets suckled by diseased sows with equaling or more than eight parities showed a significantly lower daily weight gain (DWG) compared with piglets suckled by a diseased third to seventh parity sows and diseased second parity sows (Table 7, *p* < 0.05).

#### Differences between health categories

The parity group with sows from third to seventh parity in the diseased group showed a significantly lower number of total born piglets (TBP3-7, *p* < 0.05) as well as a significantly lower number of piglets born alive (NBA3-7, *p* < 0.05). The parity group with sows of eighth or higher parity (8+) was most often characterized by significant differences. This group showed significant differences between the three health categories in the number of piglets born alive (NBA8+, *p* < 0.05). In addition, the same parity group showed a significantly higher weaning weight of the sows (SW2.8+, *p* < 0.05) in the diseased group compared with the healthy group. However, weight loss during lactation did not differ. Piglets suckled by sows in the diseased group had a significantly lower daily weight gain (DWG8+, *p* < 0.05). Additionally, first parity sows showed a significantly lower daily weight gain for the piglets suckled by sows in the diseased group, too (DWG1, *p* < 0.05).

## Discussion

The primary purpose of precision livestock farming (PLF) is to provide guaranteed, affordable, and straightforward solutions to severe problems (17). Animal welfare and farm sustainability are major concerns for future agriculture, especially pig production. Therefore, it is necessary to use more advanced methodologies such as precision livestock farming (PLF) to assist traditional farming methods (1). The NWP per sow mainly determines the profitability of piglet production. Increased litter sizes are associated with lower birth weights, lower growth rates of many light piglets (51), and lower piglet survival (31, 52, 53). Decreased survival rates and more piglets with lower performance potential make the monitoring of diseases and infections, like PDS in sows, within pig production even more crucial (23, 36, 54). It is known that infrared thermography, in general, can help to find diseased sows early on (18, 42, 43). This would allow for timely treatment. Previous studies have showed that the parity of sows influences the sows' performance (44–46). Nonetheless, little is known concerning how the parity influences the information from infrared thermography of the mammary gland. Moreover, it is largely unknown how PDS influences different parity sows. However, this knowledge is important to gain a more differentiated evaluation of the infrared thermography of the mammary gland and of its management.

### Infrared thermography and rectal temperature

#### Differences between the parity groups

Gilts showed significantly the highest temperatures on day 14 (TH14.1 and RT14.1) and at weaning (TH21.1 and RT21.1). On the other hand, the oldest sows had significantly lower temperatures (TH14.8+, RT14.8+, TH21.8+, and RT21.8+) in every health class. All temperatures (TH14, RT14, TH21, and RT21) decreased continuously from the first parity groups to the highest parity groups in every health class. Unlike shortly after farrowing, 14 days after farrowing, and at weaning, nearly all sows were without clinical disease symptoms. This is probably the explanation for the fact that temperatures 14 days after farrowing and at weaning are more age-dependent than directly after farrowing. Together with the knowledge from this study that the sows' weight increased at least up to eighth parity, this could be why the basal metabolic rate per kg body weight decreases in heavier sows. Furthermore, the relative proportion of metabolically active organs in the total body mass decreases (55). In addition, a previous study reports from heat production in sows of 400kJ/kg^0.75^ body weight (56). In relation to a 200 kg sow and a 300 kg sow, this means that the 300 kg sow has 50% more body mass, but only 35 % more heat production. This statement probably applies even more strongly today because, due to increased fertility performance, feed intake has increased in recent decades, especially during lactation (57). For this reason, large animals must consume less feed per kg of body mass than small animals; therefore, perhaps less metabolic heat is produced from feed intake. This can be an advantage when outside temperatures are high.

#### Differences between health categories

In our study, the skin temperature of the udder and rectal temperature shortly after farrowing were mainly influenced by the health status of the sows and not by age. All three health groups differed significantly with regard to TH0 and RT0. This is not dependent on parity. In addition, TH0 and RT0 were moderately correlated (see below). The second highest value, with 0.71, was detected in the group of sows with eighth and higher parity. This is in accordance with previous studies. Schmitt and O'Driscoll (19) found moderate and very strong correlations between thermographic images of the back and the ear base, as well as the rectal temperature of piglets. One study investigated sows' mammary glands by infrared thermography at 21 d, 7 d, and 1 d ante partum and 1 d, 3–4 d, and 14 d post-partum. This former study found significant correlations between the mean temperature of the first six mammary complex pairs and simultaneously measured rectal temperature (42). Another publication showed a moderate correlation between mammary skin temperature and rectal temperature (43). In our study, the rectal temperature was not affected by room temperature. For that reason, we assumed that it did not affect the animal welfare of the animals in our study.

In summary, our study and other studies confirmed a close relationship between the skin temperature of the mammary gland and other regions measured by infrared thermography and rectal temperature. Our study clearly shows that this applies to the skin temperature of the mammary gland regardless of parity. This becomes clear because the diseased groups always had the highest temperature in the thermographic image and the highest rectal temperature in our study. This means that the infrared thermography of sows' mammary glands of all parities contains clues that can help identify diseased sows, especially among old sows (correlation coefficient of 0.73 and 0.71, respectively). This is very important to know because piglets of sows with eighth and higher parity had a normal daily weight gain unless the sows became ill. When these old sows became ill, the piglets' daily weight gain was 23 % lower. Thus, if old diseased sows are identified early on, it becomes apparent which sows need to get fewer piglets by cross-fostering and which sows have to be treated. The other old sows can get as many piglets as the other parity groups. This results in increased sustainability in sow herds and, above all, increased animal welfare. For this selection, infrared thermography coupled with AI can be a helpful management tool in the future. On the other hand, it must also be said that so far, a relatively small proportion of sows on farms are needed for more than seven lactations, on the farm where the data collection for this study took place, 11%. However, this may change in the future if there are PLF-tools for the farmer so that it is easier to manage old sows.

### Correlations

TH0 and RT0 were correlated moderately. Similar moderate correlations could be seen for TH14 and RT14 in first and eighth and higher parity sows, and a fair correlation was demonstrated in second and third to seventh parity sows. Again, moderate correlations were observed between TH21 and RT21, except for TH21.2 and RT21.2. This predictability of the rectal temperature by measuring the skin temperature is in accordance with the findings of the previous studies discussed above (19, 42, 43). Otherwise, the study by Wendt et al. (58) contradicts this. However, it must be realized that they investigated the base of the ear, the back, and the anus region with infrared thermography—not the mammary gland. In addition, a more recent study contradicts this (59). The reason for this is probably the change in pig genetics over the last 20 years. Another study reported high predictability of rectal temperature by surface temperature on the snout and around the eye (21). In our study, TH14 and TH21 correlated moderately. In comparison, RT14 and RT21 correlated only fairly. Without considering the influence of parity, a previous study described similar results (18). In summary, it can be said that the surface temperature of the mammary gland is more constant from 14 days after farrowing to weaning than the rectal temperature. This is not dependent on parity. Thus, a thermal image of the mammary gland seems to provide very similar information at the two time points.

Moreover, TH0.8+ and DWG8+ correlated fairly negatively. This value supports the conclusion from chapter above. In addition, it underlines that infrared thermography of the mammary gland shortly after farrowing can indicate the expected lactation performance, especially of old sows. NWP1 and DWG1, as well as NWP2 and DWG2 correlated fairly negatively, too. This means that in litters with few piglets, higher daily weight gains tend to be expected. Therefore, it seems to be problematic to infer the lactation performance of the sows at these parities based on the daily weight gain of the piglets alone. Regarding the methodology of using only the warmest pixel of a thermal image for the evaluation, it can be said that this offers a certain risk of inaccuracies. Otherwise, a previous study (43) compared messages from the warmest pixel, from the warmest 10 and 25% of pixels from thermal images of the mammary gland and found the best message about the rectal temperature in the warmest pixel.

### Sows' and piglets' performance

#### Differences between the parity groups

In our study, the NBA of sows with parity 8–14 was significantly lower in every health class. Lavery et al. (60) compared NBA between first to sixth parity sows and found the lowest number of piglets born alive (10.9) in sixth parity sows. Higher parity sows were not investigated. Moreover, Koketsu et al. (46) reported the lowest NBA in first, eighth, ninth, and tenth parity (NBA (n): 10). Older sows were not investigated.

We found that NS8+ was significantly higher in every health class and that old diseased sows had more stillborn piglets than old sows in healthy and clinically suspicious groups. However, this difference was not significant. The reason for this probably the small number of investigated diseased sows (*n* = 11). Due to good management on the farm, there were few diseased old sows. For organic herds, Rangstrup-Christensen et al. (61) reported an increased risk for stillborn piglets for thin sows (BCS = 2) with a parity higher than four. The reason for this could be a higher risk of uterine inertia with increasing age. In addition, the documented number of stillborn piglets is partially in accordance with a previous study (62) that found an increased risk for stillborn piglets for higher parity sows when stillbirth occurrence at previous farrowing was taken into account.

NWP8+ was significantly lower in healthy and diseased sows in our study in comparison to the other parity groups. This is not in accordance with Knecht et al. (63), but the latter compared first, second, third, fourth, and fifth parity sows in winter, spring, summer, and autumn. They reported a significantly lower number of weaned piglets in autumn for first parity crossbreed sows compared with fifth parity crossbreed sows [parity 1; 9.51 ± 2.09, parity 5; 10.48 ± 1.76 (*p* < 0.05)]. Higher parity sows were not investigated. Otherwise, Lavery et al. (60) compared first to six parity sows with each other and reported that fifth and sixth parity sows weaned with ~9.9, the lowest number of piglets. The differences in the number of weaned piglets in different age groups are probably due to differences in cross-fostering. The differences, in general, are probably because we investigated modern German hyper-prolific sows that were kept under good management. Thus, in total, about three piglets more were weaned per litter in our study compared with the above mentioned studies (60, 63). As there was a high NBA in our study already in young sows, cross-fostering did not affect the NWP.

SW1.1 and SW2.1 (parity 1) were the lowest in every health class. The weight increased continuously from the first parity groups to the eighth and higher parity groups. SW1.8+ and SW2.8+ (parity ≥8) were the highest in every health class. This is not in accordance with a previous study (64) that found an increasing bodyweight for Danish sows up to fifth parity. After the fifth parity, a constant weight up to the ninth parity was observed. In the previous study, sows were weighed at the end of gestation. Higher parity sows were not investigated. Another publication (65) reported increasing weights of sows antepartum up to seventh parity. In addition, SWD1 was the highest, and SWD8+ was the lowest in every health class. This is not in accordance with the previous study (66) that compared first to fourth parity sows with each other and reported the lowest weight loss in first and fourth parity sows. However, that study is more than 20 years old, and the sows were housed in Thailand under other climatic conditions. In addition, Landrace and Yorkshire sows were investigated, but not German hyper-prolific hybrid sows.

In summary, the results of our study and previous findings show that old sows have a lower number of piglets born alive and a higher number of stillborn piglets. In our study, this was especially true for old and PDS-affected sows. The statement regarding the number of weaned piglets is not so clear, the reason for this most likely being a difference in cross-fostering. In contrast to the literature, our data show that the weight of the investigated sows increased at least up to the eighth parity, and the highest weight loss could be seen in first parity sows.

#### Differences between health categories

We found that TBP3-7, NBA3-7, NBA8+, and SW2.8+ were significantly lower in diseased groups. In addition, DWG8+ was significantly lower in the diseased group (−23%). The same could be shown with DWG1 (−13%). The two other parity groups (parity 2–7) showed no significant differences in DWG between the health classes. To our knowledge, this all has not been differentiated according to parity and health status in modern genetics. Irrespective of parity, previous studies reported similar findings but not to this quantitative extent. Two earlier studies reported that piglets that suckled by PDS-affected sows had about 5% less daily weight gain until weaning than piglets that suckled by non-diseased sows (18, 67). Patra et al. (36) showed significant differences in DWG in piglets between PDS-affected and non-diseased sows in winter (PDS-affected; 97.78 g ± 23.76, healthy; 132.25 g ± 36.1) and in summer (PDS-affected; 118.63 g ± 18.73, healthy; 141.56 g ± 30.03). Worthy of mention, this previous study focused on crossbred sows (Hampshire × Ghungroo) kept in the tropics (India). This explains the overall large difference between the DWG in piglets in our study compared with a previous study (36). We investigated German hyper-prolific sows kept under good management (about 190 g ± 30 in piglets suckled by non-diseased sows and 148–193 g ± 40 in piglets suckled by PDS-affected sows) and Patra et al. (36) investigated other crossbred sows (Hampshire × Ghungroo) in the tropics. Yu et al. (68) showed no significant differences in piglets' body weight at weaning when comparing piglets suckled by PDS-affected sows to piglets suckled by non-PDS-affected sows. However, a trend toward a negative influence of the PDS disease on the piglets' body weight at weaning could be shown.

In conclusion, old PDS-affected sows, first of all, those with eighth and higher parity, had especially lower NBA and litters with lower DWG. PDS-affected middle-aged sows (parity 3 to 7) had lower TBP and NBA and showed no significant differences in the DWG of piglets compared with healthy and clinically suspicious sows. This means that they were able to suckle as many piglets as first and second parity sows. Moreover, they were able to suckle as many piglets as those parity sows that were healthy or clinically suspicious. This knowledge is important for cross-fostering. It remains to be noted that subclinical conditions were not recognized and considered.

## Conclusion

In summary, results are suggestive that sows of higher parities (≥8) indicate a nearly normal performance after farrowing, measured in the daily weight gain of the suckling piglets, unless they become diseased. Old sows suffering from PDS show a bad performance, the disease especially having negative consequences on the daily weight gain of the suckled piglets until weaning and probably even beyond that. Infrared thermography of the mammary gland provides similar information compared to rectal temperature and can help identify diseased sows. Thus, in the future, infrared thermography of the mammary gland coupled with precision livestock farming and smart farming innovations can provide the tool to detect old diseased sows even earlier. With this knowledge, more individualized cross-fostering and more targeted piglet feeding would be possible. In this way, animal welfare for the piglets is enhanced because of better feeding and the resulting improved health. Moreover, such technology could allow the longer use of sows so that their animal welfare could also be improved, making it possible for the farm to save on herd replacement costs.

## Data availability statement

The original contributions presented in the study are included in the article/supplementary material, further inquiries can be directed to the corresponding author/s.

## Ethics statement

The animal study was reviewed and approved by Animal Welfare Officer of the University of Veterinary Medicine Hannover, Foundation, Hannover, reference: TVO-2020-V-9. Written informed consent was obtained from the owners for the participation of their animals in this study.

## Author contributions

MW, HH, AD, JT, IT, and CV conceptualized the study and acquired funding for the project. SR, L-ST, MW, and CV designed the methodology. SR, MW, and CV validated the study. SR, BC, and CV performed the formal analysis. SR investigated the experiment and prepared the original manuscript draft. SR, HH, and CV performed the data curation. SR, BC, MW, and CV contributed to the writing—review and editing. SR and CV visualized the study. MW and CV supervised the project. AD administered the project. All authors have read and agreed to the published version of the manuscript.

## Funding

This research was funded by EIP-Agri (European Innovation Partnership Agricultural Productivity and Sustainability) and European Agricultural Fund for Rural Development. This Open Access publication was funded by the Deutsche Forschungsgemeinschaft (DFG, German Research Foundation) within the programme LE 824/10-1 Open Access Publication Costs and the University of Veterinary Medicine Hannover Foundation.

## Conflict of interest

Author AD was employed by EVH Select GmbH, Meppen, Germany. Author HH was employed by BHZP GmbH, Dahlenburg-Ellringen, Germany. The remaining authors declare that the research was conducted in the absence of any commercial or financial relationships that could be construed as a potential conflict of interest.

## Publisher's note

All claims expressed in this article are solely those of the authors and do not necessarily represent those of their affiliated organizations, or those of the publisher, the editors and the reviewers. Any product that may be evaluated in this article, or claim that may be made by its manufacturer, is not guaranteed or endorsed by the publisher.
